# Adapting a nurse-led primary care initiative to cardiovascular disease control in Ghana: a qualitative study

**DOI:** 10.1186/s12889-020-08529-4

**Published:** 2020-05-24

**Authors:** Leah A. Haykin, Jordan A. Francke, Aurelia Abapali, Elliasu Yakubu, Edith Dambayi, Elizabeth F. Jackson, Raymond Aborigo, Denis Awuni, Engelbert A. Nonterah, Abraham R. Oduro, Ayaga A. Bawah, James F. Phillips, David J. Heller

**Affiliations:** 1 Arnhold Institute for Global Health, Icahn School of Medicine at Mount Sinai, 1216 5th Avenue, New York, NY 10029 USA; 2 Navrongo Health Research Centre, Navrongo, Ghana; 3 Heilbrunn Department of Population and Family Health, Columbia University Mailman School of Public Health, New York, NY 10032 USA; 4 Regional Institute for Population Studies, University of Ghana, Legon, Ghana

**Keywords:** Cardiovascular disease, Ghana, Community health

## Abstract

**Background:**

Cardiovascular Disease (CVD) is a growing cause of morbidity and mortality in Ghana, where rural primary health care is provided mainly by the Community-based Health Planning and Services (CHPS) initiative. CHPS locates nurses in community-level clinics for basic curative and preventive health services and provides home and outreach services. But CHPS currently lacks capacity to screen for or treat CVD and its risk factors.

**Methods:**

In two rural districts, we conducted in-depth interviews with 21 nurses and 10 nurse supervisors to identify factors constraining or facilitating CVD screening and treatment. Audio recordings were transcribed, coded for content, and analyzed for key themes.

**Results:**

Respondents emphasized three themes: community demand for CVD care; community access to CVD care; and provider capacity to render CVD care. Nurses and supervisors noted that community members were often unaware of CVD, despite high reported prevalence of risk factors. Community members were unable to travel for care or afford treatment once diagnosed. Nurses lacked relevant training and medications for treating conditions such as hypertension. Respondents recognized the importance of CVD care, expressed interest in acquiring further training, and emphasized the need to improve ancillary support for primary care operations.

**Conclusions:**

CHPS staff expressed multiple constraints to CVD care, but also cited actions to address them: CVD-focused training, provision of essential equipment and pharmaceuticals, community education campaigns, and referral and outreach transportation equipment. Results attest to the need for trial of these interventions to assess their impact on CVD risk factors such as hypertension, depression, and alcohol abuse.

## Background

CVD is the leading global cause of morbidity and mortality [1, 2], and the prevalence of CVD is rising in low- and middle-income countries (LMIC) [3–6]. Non-communicable diseases (NCDs) like CVD caused 70% of all global deaths in 2017, with CVD the leading contributor [2, 7]. These increases are due to demographic, epidemiologic, and nutritional transitions caused by the development and urbanization of LMICs [8]. Consequently, the burden of CVD is most acute in LMICs, where some 74% of global CVD morbidity and mortality occurs [1, 9]. Moreover, in both high- and low-income countries, CVD and other NCDs disproportionately affect the lowest-income populations [10]. In many LMICs, weak health infrastructure further undermines control of these conditions [11].

Recognizing the detrimental impact of NCDs on human health and sustainable development [6, 12], the World Health Organization (WHO) has developed strategies for their control, such as the 2010 Package of Essential NCD Interventions (WHO-PEN) [13]. In 2013, the WHO released a Global Action Plan for the Prevention and Control of NCDs, with a goal to achieve a 25% relative reduction in worldwide premature mortality from the estimated prevalence of NCDs in 2013 by 2025, via six action objectives [14]. Objective 4 of this Global Action Plan calls for adapting existing health systems by improving NCD primary health care prevention and services. The WHO next released the HEARTS intervention package in 2016, which builds on WHO-PEN by detailing cost-effective interventions to prevent and treat CVD and its risk factors using community-based primary health workers [15, 16].

This global agenda is particularly relevant in sub-Saharan Africa, where CVD prevalence is increasing dramatically [17, 18]. Adjusted for age, West African countries such as Ghana have among the highest prevalence of CVD and its risk factors globally [19]. Hypertension, for example, affects up to 32% of adults in Ghana [20] and 24.5% of individuals in the Kassena-Nankana East and West districts [21]. The rising tide of CVD in this region has prompted several initiatives to implement select elements of the WHO-PEN and HEARTS protocols [22, 23], including for the control of hypertension in Ghana [24]. However, none have attempted to build the complete HEARTS CVD care model into an existing primary care system.

In regions where physicians are scarce, non-physician health workers (NPHWs) including nurses, pharmacists, and community health officers (CHOs) can effectively provide primary care [25, 26]. In Ghana, which has fewer than one physician per 10,000 people [27], the national CHPS program is staffed by CHOs, community health nurses who have been trained for 18 months in the provision of primary health care services and subsequently oriented for 6 months in community engagement and outreach. Although these CHOs practice predominantly at CHPS clinic compounds, most are also responsible for home visits and community health education.

CHPS’ model for community-level primary care in Ghana is the outcome of a process of implementation science that commenced in 1994 and continues to the present [28–32]. Following the 1978 Declaration of Alma Ata, the Ghanaian government embraced the goal of “Health for All by the Year 2000,” setting out to address primary causes of death and disability, such as infectious diseases of children under age five [33]. To develop a strategy for addressing the “Health for All” goal, an 18 month, three village participatory pilot study was convened to explore culturally compatible means of nurse deployment and support [29, 32]. Based on results of the pilot, a factorial experiment was convened that involved redeploying nurses from sub-district and district clinics to live and work in satellite Community Health Compounds (CHCs) located in remote rural communities and provide door-to-door health screening and primary care to community members as CHOs [34]. Launched as a district-wide trial of the Navrongo Health Research Centre (NHRC), results showed that nurse deployment could reduce childhood mortality by 50% in only 3 years [34], which led to a national policy to scale-up the Navrongo model in nearly 4500 CHCs that are dispersed across all districts in Ghana [31, 34, 35]. CHPS operations are supervised by sub-district leaders (SDLs), who are midwives accountable to the Ghana Health Service. Since its inception as a national program in 2000, implementation science has been directed to testing replicability [31], accelerating the pace of scale-up [36], developing emergency referral services [37], improving family planning effectiveness [38], and improving systems support for CHPS operations [36]. The current study is a component of a more general implementation science agenda for improving CHPS functionality and effectiveness [39].

However, further reform and development of CHPS is needed. Since the conception of CHPS, CVD has emerged as a major cause of morbidity and mortality in Ghana [40, 41], and CHPS compounds designed primarily for management of maternal health and childhood illnesses remain poorly equipped to respond to this epidemic. Moreover, the feasibility of leveraging the CHPS model of nurse-led preventive and curative primary care for CVD and other NCDs has yet to be established as a coherent component of their work.

Recent work demonstrates that NPHWs are effective in the assessment and treatment of CVD and other NCDs. CHOs in multiple settings can screen patients for CVD risk with similar accuracy to nurses and doctors [42], which can improve identification of patients at high risk compared to usual care [43]. Furthermore, recent systematic reviews demonstrate that NPHWs can prevent and treat CVD by prescribing medications for risk factors such as hypertension and diabetes [44–46]. Our study seeks to explore perceptions of NPHWs regarding their capacity to manage CVD and barriers to implementing the WHO HEARTS package at CHPS facilities in the Upper East Region (UER) of Ghana. Presently, CHPS nurses do not manage CVD, despite working in areas where CVD risk factors such as hypertension [21] and obesity [41] are increasingly common.

## Methods

### Study design and theoretical framework

This qualitative research is a component of a mixed-methods implementation science study that aims to develop a protocol for leveraging CHPS nurses to provide CVD care through the WHO-PEN protocol. Our previous work aimed to quantify and trend the burden of CVD mortality among adults in the UER as a function of age, gender, and socioeconomic status to identify high-risk groups [47]. We then undertook the current qualitative evaluation of barriers to provision and acquisition of CVD care at CHPS facilities to inform the practical design of a modified CVD prevention program in this region.

Due to the lack of baseline data on perceptions of CVD in this population, we employed grounded theory to identify codes and themes. Briefly, grounded theory expressly avoids a priori theoretical frameworks for analyzing data, and instead involves collecting data first and then subsequently building codes and themes “grounded in data systemically gathered and analyzed.” [48]. Specifically, we employed a situational analysis approach to grounded theory [49], reviewing these data not as specific elements causing or effecting each other but rather as a contextual whole. This approach allowed our research team to theorize based on the data during and after its analysis, rather than employing an a priori theoretical framework. It also allowed us to examine the data as a whole (its entire discourse, as well as specific statements) [49] to shape theme development and analysis, rather than imposing our own preexisting beliefs and assumptions on individual fragments or narratives.

Because this analytic approach focused heavily on social and cultural context (in addition to logistical and biomedical factors), the social-ecological model [50] emerged as optimal for framing these data and their underlying themes. This theoretical framework seeks to understand the multifaceted and interrelated effects of individual and environmental factors, in order to identify opportunities for health promotion [50]. This model aligned best with use of situational analysis because it acknowledges the multiple levels at which societal (and other situational) factors influence individual beliefs and behavior, such as interpersonal, institutional, community, and sociocultural factors, many of which relate to provider and patient beliefs on NCD control in low-income settings [51, 52].

### Sample

We conducted our study in the Kassena-Nankana East and West districts in the UER of Ghana. Study participants were NPHWs working in the CHC setting. Data were collected between April and August 2017. In-depth interviews (IDIs) were conducted at CHCs, and the providers sampled were CHOs and SDLs.

### Recruitment and data collection

We used convenience and purposive sampling to select CHOs and SDLs from CHCs throughout the study area. We purposefully contacted respondents across a geographically diverse list of CHPS locations to minimize bias. We excluded providers who were not fluent in English. We recruited NPHWs through letters to the Kassena-Nankana East and West districts’ health directorate and SDLs, as well as through a stakeholder engagement meeting at the NHRC, both of which described the aims of the study to prospective participants. We planned to recruit 20 NPHWs to allow for adequate data saturation [53, 54], as previous work suggests that new themes emerge infrequently after analysis of twelve qualitative interviews, and that these subsequent themes tend to be variations on existing themes rather than novel ones [55].

Researchers include research staff at the NHRC with experience living and conducting field research in the community served [AA, EY, ED, RA, DA, EN, AO], researchers at the University of Ghana [AB], a physician and medical students at the Icahn School of Medicine at Mount Sinai [DH, LH, JF], and researchers at Columbia University [EJ, JP]. Both female [LH, AA, ED, EJ] and male [JF, EY, RA, DA, EN, AO, AB, DH] researchers participated in the study. All participants had baccalaureate degrees, and several had additional professional or graduate degrees.

The semi-structured IDI guides were designed to evaluate providers’ clinical experiences with CVD, barriers to CVD care, training of CHOs, health literacy, community CVD burden, CVD risk factors, CVD prevention and treatment, CHPS resources, and CHO capabilities, as well as their suggestions for improvement. The final IDI guides can be found in the appendices.

Researchers [LH, JF, AA, EY, ED] conducted the interviews in English after obtaining written consent. Care was taken to ensure privacy to allow participants to speak freely and to reduce social desirability bias. We supplemented the IDI guides with questions based upon respondents’ initial responses. No repeat interviews were performed, and no field notes were taken. The IDIs, which ranged from 19 to 49 min in length, were audio recorded. Members of the research team transcribed the IDIs, with the quality of transcripts checked by other members of the team. Transcripts were corrected only to better adhere to the content of the audio recordings. Data were deidentified prior to analysis.

### Analysis

We developed codes and themes for the study by iterative review, rooted in the grounded theory approach detailed above [48]. Multiple reviewers [LH, JF] separately reviewed initial transcripts, individually introducing codes. They then met to compare these codes with other reviewers [RA, EJ, DH] in order to agree upon which existing codes best captured the data and to create new codes jointly in order to classify uncoded content, representing constant comparative analysis [48]. After a set of 15 codes emerged, reviewers coded all transcripts. Reviewers [LH, DH] then examined these codes to identify emerging themes and analyze the broader implications of these themes [56]. As described above, our analysis employed the social ecological model, allowing for exploration of the interrelationships between the experiences of individual providers and their broader environmental contexts. In accordance with grounded theory, we avoided assigning this or any other model or framework to the data until after coding was complete. We conducted all aspects of study design, data-gathering, analysis, and results reporting as per the Consolidated Criteria for Reporting Qualitative Research [57] (Supplemental File 1). We performed all analysis using NVIVO software (version 11) [58].

## Results

**Fig. 1:**
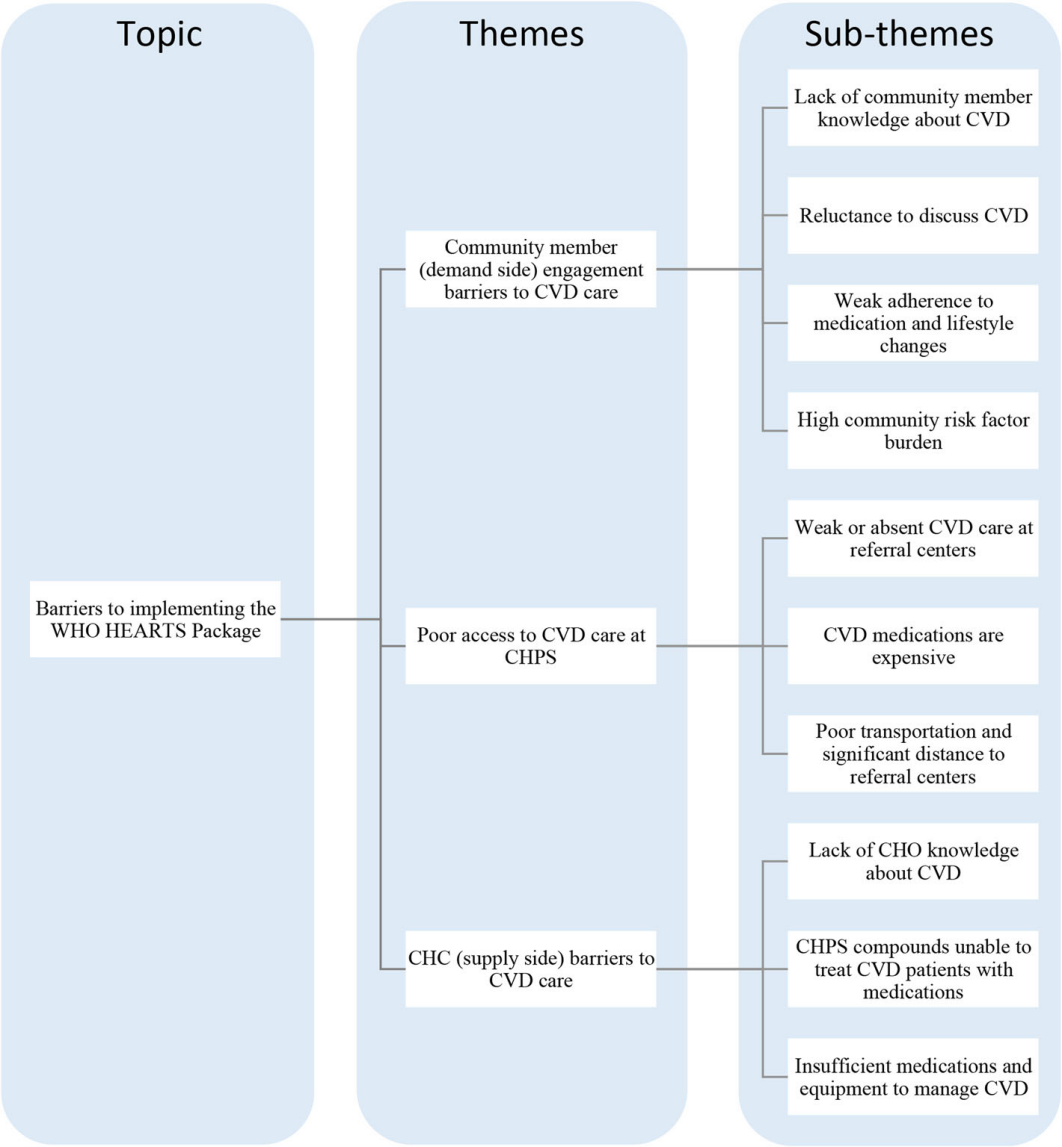
Flowsheet: Thematic Analysis

Our sample included 21 CHOs and 10 SDLs, for a total of 31 participants across 23 CHPS sub-districts. Respondents discussed barriers to CVD care – and solutions to these challenges – across three themes: community demand for CVD care; community access to CVD care; and provider capacitity to render CVD care. A flowsheet (Fig. 1) depicts as a tree these three themes as well as ten sub-themes – each directly informed by our (fifteen) main codes and their sub-codes. Themes and sub-themes were grounded in the content of the IDIs via those codes, developed in turn by the constant comparative analysis of data detailed above. All analysts found that the sample achieved a high degree of thematic saturation.

### I. Community member (demand-side) engagement barriers to CVD care

This theme refers to challenges NPHWs face with rendering CVD care. These challenges include high risk factor burdens, poor health literacy, and other community member factors.

Many participants believe that the communities that they serve experience a high burden of CVD risk factors. These risk factors include stress, alcohol and tobacco use, diet, and sedentary lifestyle.

Most SDLs say that stress, particularly family-related stress, is a leading cause of CVD in their communities. Participants pointed to several common stressors, such as family problems, work, overthinking, and emotional triggers. Common family problems included being “abandoned” when one’s children move to cities, being childless and unable to support oneself, having children who are “useless,” bad marriages where one is commonly insulted, and not being able to afford school tuition for children. Respondents spoke often about stress due to the migration of youth to nearby cities and towns. This has created an unprecedented problem in rural Ghana as traditional support for the elderly has declined. These social dynamics are described by an SDL:

*“Some people have got a lot of problems.… Like you have delivered your children, all of them have run away and left you alone. You are old, and you are still doing everything for yourself. You see, that is a problem that can give you heart disease. And at times, some people don’t even have children. If you don’t have a child, how do, you see, you are old, and you needed somebody to support you, there is nobody to support you. So that, all that will lead to heart disease.” (SDL)*

Participants were also worried about the degree of alcohol consumption that they see in their communities, and its implications for CVD and community health more generally. As one SDL describes,

*“To me, the biggest one is the alcoholism…. Anywhere you move you see that everyone is just drunk, drunk, drunk.” (SDL)*

This concern was compounded by the belief that patients are not forthcoming in speaking about their alcohol use with CHOs. Some CHOs have attempted to intervene on patients who use alcohol excessively, either by creating a therapeutic alliance with them or by involving their families, with varying levels of success.

Respondents reported that many community members smoke tobacco, and that this behavior is particularly common among men and youths:

*“This is a tobacco farming community, and we all know that smoking is a very high-risk factor. And almost all the youth in this community smoke excessively, so actually it’s a problem here. The smoking. Almost all the youth, even teenagers, smoke here.” (CHO)*

Several CHOs say that they are encouraged by their SDLs to counsel patients regarding the risks associated with smoking and the benefits of smoking cessation. Some CHOs found, however, that patients often try to conceal their smoking status from NPHWs.

Finally, numerous participants feel that community members’ diets and sedentary lifestyles put them at high risk for CVD. The diets described were heavy in salt, fat, meat, and soda, and deficient in vegetables. Some respondents say that they often counsel patients on the benefits of diet and exercise. One SDL describes that these conversations can be difficult, however, due to conflicting cultural attitudes regarding diet, exercise, and weight:

*“We don’t see the need to exercise. We don’t see the need to diet. You feel that growing big is something that is good in our society, that’s what they think. When you are big, it’s a sign of good living.” (SDL)*

In addition to having many CVD risk factors, a preponderance of participants feel that community members lack a basic understanding of the symptoms and health consequences of CVD. As a result, individuals who develop CVD symptoms sometimes eschew care and as several respondents describe, instead attribute these symptoms to other diseases, such as malaria, or to divine judgment. Many note that due to a combination of these factors, individuals who are afflicted with CVD are often unaware of their illness.

Medical screening of asymptomatic individuals for NCDs is exceedingly rare in Ghana, which likely contributes to this low level of health literacy. In fact, many community members are unfamiliar with the concept of preventative screenings and asymptomatic treatment. This lack of familiarity was described by numerous respondents:

*“It’s lack of knowledge…. If you do not visit them, they don’t know what check-ups are…. So, they sit at home and then develop the pressure without managing it.” (CHO)*

*“Some of them, when it is better, they don’t take the medication. That is one problem we can face. They don’t take the medication because they think they are fine.” (CHO)*

Several respondents note that once diagnosed with CVD, community members do not feel comfortable discussing their health problems, representing a barrier to care. Participants mentioned patients who do not inform friends and family of their CVD because they do not want to burden them with the costs of their treatment. Others do not want to discuss their CVD because they do not understand the serious implications of their illness. Some CHOs also mentioned stigma surrounding CVD:

*“They think when they share it out and you also tell others. Maybe this house gets to know that that house has someone suffering from this condition it means they will lose respect or something like that, so they will not even like others to know.” (CHO)*

Once connected to care, some patients demonstrate poor adherence to CVD treatment. CHOs routinely survey medication adherence both at CHPS compounds and on home visits. They say that community members often become ill because they discontinue their medications when they are feeling better. However, the reasons for community members’ poor adherence are likely multifactorial, including fear of potential side effects, cost, misunderstanding, polypharmacy, and lack of symptoms [59].

### II. Poor access to CVD care at CHPS

This theme refers to community members’ difficulty obtaining care for CVD. Respondents described difficulty due to cost, distance, and other barriers to reaching a CHPS site, independent of the logistics of CHPS itself.

CHPS workers say that they most often encounter heart disease patients on home visits, because these patients generally do not come to CHPS to receive care. As described by a CHO:

*“[T]hey don’t come into the facility with that condition. Unless when we do home visiting and we identify them.” (CHO)*

Several participants said that patients with heart disease used to come to CHPS compounds more often. However, they do not anymore, because CHPS compounds no longer carry CVD medications.

*“Some of the treatments for the other diseases, we don’t have them here. So, somebody will say ‘Why do I waste my time to come to this clinic, come and expose myself to them, after all they are not going to give me any treatment.’.... So, it’s better that I go to Navrongo than come to this facility.” (CHO)*

At present, CVD care is largely provided at referral centers. However, patients experience barriers to receiving care at these centers. Referral centers are often far away from the communities in which patients live; even when geographically close, many patients lack the means of transport to get there. Some patients, particularly the elderly, require help getting to referral centers, but do not have anyone to accompany them.

*“Especially, the elderly one. Those who don’t also have someone to support them…. So, if the person cannot walk to Navrongo, there is no money to transport to Navrongo, so the patient will not go.” (CHO)*

Cost is a large barrier to receiving care at referral centers. Many patients will pay others for the fuel or vehicle needed for transport. But as referral centers often draw from low resource areas, this cost can be too great for some, as outlined by numerous participants:

*“Sirigu is nearer, but before the person even gets there, maybe that person will need a motor rider, that person will need fuel, and that person has no money. And to talk of Navrongo, when you mention Navrongo, they even get frightened. So actually, it’s very difficult for them.” (SDL)*

*“You know when she came, and I mentioned referral, the mother went back to the house…. I’m sure they have to go to the market to look for the money, but when they get there, by all means, they will not buy medicines, they will have to eat and other things. So, the poverty level here is very high so when you are referring.” (SDL)*

*“You see, this village like this is a deprived village. They don’t have money. So, some of them, it is really a problem to them.” (CHO)*

Respondents describe significant barriers to accessing CVD care, namely CHPS-related limitations, lack of transportation, distance to referral centers, and high costs. As a result of these barriers, many patients with CVD or CVD risk factors such as hypertension are not able to successfully access care.

### III. Community health center (supply-side) barriers to CVD care

This theme referred to challenges that CHOs and the CHPS program face with rendering CVD care. We found that these challenges include gaps in training, logistics, and other structural factors.

While respondents highlighted strengths with regards to the CHOs’ training, most participants, particularly SDLs, expressed that CHOs lack important knowledge about CVD. CHOs were confident in their knowledge of topics such as CVD risk factors and detection. However, in a quiz administered at 21 CHCs, CHOs showed gaps in knowledge about CVD risk factors and causes, as well as about the diagnosis and treatment of CVD.

Due to CHPS’ origins as an organization primarily focused on improving maternal and child health, CVD has not historically been at the core of CHO training. During their schooling, CHOs receive training about CVD that focuses primarily on prevention and diagnosis. However, many SDLs felt that this training is insufficient. Further, many CHOs expressed that they had not learned enough about CVD during their training. This sentiment was expressed by an SDL:

*“[CHO knowledge of heart disease symptoms is] not adequate per my own assessment. Because some of them, they can’t even tell you the treatment you give to maybe a patient with high blood pressure, diabetic, stroke.” (SDL).*

Contributing to these knowledge gaps is lack of exposure. Participants felt that knowledge gained during training is lost over time because CHOs do not often see CVD patients at CHPS facilities.

*“After school scarcely will you open the book again…. But with the refresher training, it reminds you of the things you have forgotten, then you are refreshed to do the work better.” (CHO)*

As such, almost every CHO said that more in-service trainings would help them better understand and care for CVD. Participants felt that trainings should focus on CVD prevention, symptoms, management, and treatment.

In addition to enhanced training, participants expressed that they also require materials including medications, blood pressure monitors, motorbikes, and fuel in order to provide adequate care for CVD and its risk factors at CHPS compounds. Presently, most CHPS compounds do not carry essential medications for CVD risk factors such hypertension, hyperlipidemia, and diabetes. This is because the National Health Insurance Scheme (NHIS) does not reimburse CHPS compounds for disbursing medications such as antihypertensives, statins, and antihyperglycemics. As a result, patients are forced to travel to larger regional health centers for treatment. As outlined previously, because these facilities are farther away and transportation to them is poor, patients often choose not to go, and instead live with untreated CVD or CVD risk factors. For these reasons, participants feel that NHIS’ decision to not reimburse CHPS for these medications is making their communities sicker.

While CHOs may not dispense medications, they are currently able to screen for, diagnose, monitor, and manage straightforward cases of CVD, as well as to refer to regional health centers. Some participants felt that this level of care was not adequate to help patients with conditions such as hypertension. Others say that they turn CVD patients away because they know that they are unable to treat them. At times, patients do not understand the limitations placed on CHPS compounds, and this can be discouraging for CHOs. As one CHO states,

*“They ask you questions like, ‘Now you come and stand and talk like this. How come when I tell you people, well I’m having BP, my drugs, you tell me I should go to hospital? Then what are you people doing?’ You see that? So sometimes those things discourage us. We feel bad. We just wish that we could be able to do it.” (CHO)*

Most CHOs want to be able to treat CVD pharmacologically at CHPS compounds. Many participants felt that with more training, CHOs would be capable of treating straightforward CVD cases with medications. They feel that this effort would be beneficial to patients by making these medications more accessible.

*“They can treat by sometimes giving some of the health centers, the smaller clinics, the opportunity to also treat…. Because some of us, we don’t have access to treatments. We only monitor, refer. And here, like this one, you tell them to go to Navrongo, it’s like you cursed them or something. They don’t want to go.” (CHO)*

Many CHOs want a return to the “old system” in which NHIS reimbursed CHPS compounds for CVD medications. They feel that this would reduce the burden on low-income patients and increase medication adherence.

*“Most of the hypertensive cases who are put on drugs as they grow older, some today is on the drug, tomorrow the person is not. When asked why, they go ‘Oh, I don’t have money to buy.’ But if GHS is able to come out with drugs, that will cover health insurance, people will get with it.” (CHO)*

In addition to medications, most participants claimed that their facilities lack equipment and materials necessary for managing CVD. Notably, they held that more blood pressure apparatuses are needed for adequate care. Several CHOs reported one apparatus was available at their facility; some CHPS compounds had none. Their absence prevents routine blood pressure surveillance during outreach and home visits, necessitating referral of patients to hospitals or CHCs for routine blood pressure readings. Non-adherence with such referrals thus impedes the provision of even the most basic of CVD interventions, as described by a SDL:

*“Even to go around with the common BP apparatus, they don’t have. For the facility, we have one and it breaks down very often. So, they don’t have. They only refer per symptoms…. It’s the BP apparatus that you use to measure, and if it’s broken down, you can’t tell whether the person’s BP has risen, whether it’s gone down.” (SDL)*

Participants also lack the motorbikes and fuel required to go on home visits and to take patients to the hospital. Some say that due to NHIS underfunding, they currently use their personal motorbikes and fuel to transport patients, because many patients cannot afford their own transport:

*“We go into the communities such cases, we interact with people, attend to old, the aged and all those things. Now we don’t have motor bikes, we don’t have fuel…. These are some of the things hampering the work. If those things are improved, it will go a long way to help.” (CHO)*

## Discussion

To our knowledge, our study is the first to explore the qualitatively perceptions of nurses within the CHPS program regarding implementation of the HEARTS protocol in Ghana. Our use of IDIs allowed for a nuanced understanding of barriers to implementing the HEARTS package, which include inadequate CHO training; poor health literacy among community members; lack of access to CVD medications and other necessary resources; a high burden of CVD risk factors; and difficulty accessing CHPS compounds and referral centers. Although previous pilot studies have implemented elements of the HEARTS protocol through CHPS [24], our work suggests means to adapt CHPS itself from a program primarily focused on infectious disease and child and maternal health to that which functions across the lifespan to accommodate all aspects of this integrated CVD care model, as well as the care of other NCDs. These approaches include, among others, improved 1) access to essential medications; 2) provider training; 3) community member education; and 4) means of transportation to referral sites, as detailed below. While this study specifically examined the perceptions of NPHWs in the UER of Ghana, its findings regarding barriers and facilitators to implementing the HEARTS package have implications for other LMICs and low-resource settings. This work also supports a growing body of evidence [42–45] that the scope of NPHWs’ practice can effectively be expanded to address CVD and other NCDs in areas of physician scarcity, building on the demonstrated efficacy of NPHWs in treating and preventing infectious diseases [60–62].

CHPS’ current infrastructure struggles to provide the 23 medications that HEARTS considers essential for primary care CVD interventions [16], in large part due to lack of timely reimbursement by the NHIS; furthermore, CHOs are prohibited from prescribing them when available. However, CHOs expressed confidence in their capacity to prescribe and monitor patients taking these medications, consistent with evidence from other contexts that nurses can safely render such care [63–66]. Abdel-All et al. (2017) found that training was effective in raising pre-training scores in assessments of knowledge about CVD, with moderate decline in scores 6 to 8 months post-training, which could be minimized with refresher trainings [63]. The HEARTS package provides evidence-based resources that should be used to design such trainings [16]. Further work should test this hypothesis by permitting CHOs to prescribe hypertension medications under physician oversight. Most respondents also feel that their facilities also require more BP cuffs, motorbikes, and fuel, for which the HEARTS package suggests potential funding sources [16].

Although CHOs and SDLs voiced concern over community members’ lack of awareness of CVD risk factors despite a high community CVD burden, they expressed optimism that individual counseling coupled with community durbars, radio broadcasts, and informative posters in local dialects could disburse health information. The HEARTS package provides a protocol that can be adapted to guide individual counseling, durbars, and other modes of communication to counsel patients on behaviors that promote cardiovascular health [16].

The distance and cost associated with travel to referral centers are major barriers to seeking care for many patients with CVD. Atuoye et al. (2015) underscore the need for public policy to address rural transport problems in Ghana in order to improve health and call for sustainable transport services driven by community participation [67, 68]. Access to hospital-level CVD management could also be increased through further expansion of the National Ambulance Service [69].

Lastly, CHOs and SDLs spoke to the high prevalence of CVD risk factors such as stress, alcohol use, smoking, poor diet, and sedentary lifestyle in their communities. Each of these risk factors contributes to multiple other chronic conditions: for example, anxiety is highly associated with depression, musculoskeletal pain, and gastrointestinal disorders in addition to CVD [70]; alcohol and other substance abuse similarly contributes to each of these conditions. Future work to target these underlying CVD risk factors through CHPS using the approaches proposed in this paper – namely improved access to medications, provider training, community member education, and transportation – could therefore have downstream effects on other diseases processes as well. Previous interventions in Ghana have addressed CVD risk factors in isolation, such as hypertension, but none have successfully impacted multiple risk factors [24, 46]. A pilot initiative to counsel vulnerable community members on these factors – for example, through counseling on alcohol and tobacco cessation – could therefore impact outcomes such as depression in addition to blood pressure control, especially if supplemented by medication, community outreach, and improved care accessibility as described above. To this end, we are currently developing an intervention based on the results of this study involving the joint screening and treatment of depression and hypertension by NPHWs within the CHPS system. This work could generate models for integrated chronic disease care beyond the HEARTS protocol, as a framework potentially adaptable to contexts outside the CHPS model.

Our approach has several limitations. While we sought to minimize bias in interview analysis through codes derived from the independent agreement of four analysts and validated by achievement of thematic saturation, our findings are subjective given the qualitative nature of this study. Additionally, our data analysts and several interviewers were external observers from the United States with prior beliefs regarding CVD care – especially regarding CVD treatment and risk factor management – that may have biased interpretation. Although all participants spoke English, not all learned English as a first language, and as such, language barriers may have impeded the accurate gathering of data. As this study represents only 31 participants from across the Kassena Nankana East and West districts, NPHWs who were not interviewed may have differing perceptions that were not captured. While NPHWs from all CHCs in the Kassena Nankana East and West districts were contacted for participation in this study, the group that chose to participate may non-randomly differ from non-participants. We sought to ask broad, open-ended questions about CVD care and its barriers, but our interviews omitted some potentially relevant topics, such as how CVD care was previously provided in the region, the role of health volunteers in providing counseling about CVD, and community members’ behaviors related to CVD. Nevertheless, our findings represent an important first evaluation of the perception of NPHWs regarding barriers to implementing the WHO HEARTS package at CHPS facilities in the UER of Ghana.

## Conclusion

We found that NPHWs in the UER of Ghana hold many strong beliefs regarding how to reform the CHPS program to provide care for CVD. While NPHWs voiced many concerns regarding obstacles to providing such care, they also listed several feasible interventions to address them, including enhanced training of CHOs and education of community members about CVD, more accessible methods for disbursement of CVD medications, increased funding for equipment, and improved means of transportation to referral sites – each of which could improve CHPS’ care for CVD as well as other chronic diseases. Although further formative work to develop these solutions remains, an intervention to address these gaps in care could constitute a novel step towards adapting HEARTS to the CHPS model in the UER of Ghana.

## Supplementary information

**Additional file 1.** COREQ (COnsolidated criteria for REporting Qualitative research) Checklist.

## Data Availability

The deidentified IDI transcripts with or without coding generated during this study are available upon request to the corresponding author.

## Abbreviations

CHC: Community health compound
CHO: Community health officer
CHPS: Community-Based Health Planning and Services
CVD: Cardiovascular disease
IDI: In-depth interview
LMIC: Low- and middle-income country
NCD: Non-communicable disease
NHIS: National Health Insurance Scheme
NHRC: Navrongo Health Research Centre
NPHW: Non-physician health worker
PEN: Package of Essential Non-Communicable Disease Interventions
SDL: Sub-district leader
UER: Upper East Region
WHO: World Health Organization
